# Molecular Mechanism of Switching of TrkA/p75^NTR^ Signaling in Monocrotophos Induced Neurotoxicity

**DOI:** 10.1038/srep14038

**Published:** 2015-09-15

**Authors:** Vivek Kumar, Amit Kumar Gupta, Rajendra Kumar Shukla, Vinay Kumar Tripathi, Sadaf Jahan, Ankita Pandey, Akriti Srivastava, Megha Agrawal, Sanjay Yadav, Vinay Kumar Khanna, Aditya Bhushan Pant

**Affiliations:** 1 CSIR-Indian Institute of Toxicology Research, Lucknow-226001, India; 2 CSIR-Central Drug Research Institute, Lucknow-226001, India.

## Abstract

We demonstrate the role of molecular switching of TrkA/p75^NTR^ signaling cascade in organophosphate pesticide-Monocrotophos (MCP) induced neurotoxicity in stem cell derived cholinergic neurons and in rat brain. Our *in-silico* studies reveal that MCP followed the similar pattern of binding as staurosporine and AG-879 (known inhibitors of TrkA) with TrkA protein (PDB ID: 4AOJ) at the ATP binding sites. This binding of MCP to TrkA led to the conformational change in this protein and triggers the cell death cascades. The *in-silico* findings are validated by observing the down regulated levels of phosphorylated TrkA and its downstream molecules viz., pERK1/2, pAkt and pCREB in MCP-exposed cells. We observe that these MCP induced alterations in pTrkA and downstream signaling molecules are found to be associated with apoptosis and injury to neurons. The down-regulation of TrkA could be linked to increased p75^NTR^. The *in-vitro* studies could be correlated in the rat model. The switching of TrkA/p75^NTR^ signaling plays a central role in MCP-induced neural injury in rBNSCs and behavioral changes in exposed rats. Our studies significantly advance the understanding of the switching of TrkA/p75^NTR^ that may pave the way for the application of TrkA inducer/p75^NTR^ inhibitor for potential therapeutic intervention in various neurodegenerative disorders.

Neurogenesis plays a critical role in the maintenance of the embryonic and adult brain, while alterations in this process lead to progression of neurodegenerative diseases^1^. Signaling through neurotrophic receptors is necessary for promoting neurogenesis, cellular development, survival and functional maintenance of neurons^2^. Neurotrophic factors such as nerve growth factor (NGF) binds to TrkA, and brain derived neurotrophic factor (BDNF) and neurotrophic factor 4 (NT4) bind to TrkB, while neurotrophic factor 3 (NT3) binds to TrkC^3^. Binding of neurotrophic factors with Trk receptors results in their dimerization and autophosphorylation, which triggers cell proliferation signaling cascades including PI3K/Akt, and Ras/Raf/MEK1/2/ ERK1/2 Map kinases^2^. These signals prevent neural cell injuries and induce cell proliferation, differentiation via activation of transcriptional factor CREB and up-regulation of anti-apoptotic BCl_2_ protein^4^. In addition, NGF also interacts with p75 neurotrophin receptor (p75^NTR^) with lower affinity, which is a membrane protein belonging to the TNF receptor superfamily^5^. In the presence of TrkA receptor, p75^NTR^ facilitates high affinity binding of NGF and activation of cell survival pathways, while, in the absence of TrkA receptor, p75^NTR^ induces cell death pathways^6^. In Alzheimer’s disease (AD), the level of pro-NGF increases due to down regulation of TrkA, thus the level of pro-NGF is high in patients suffering from AD as it is considered to mediate a pro-apoptosis pathway via activation of p75^NTR^ 7,8,9. Few studies have reported that pro-NGF inhibits NGF mediated TrkA activation in PC12 cells^10^. The derivatives of organic mercury compounds such as thimerosal and methyl mercury have been found to be linked with decreasing the process of TrkA autophosphorylation and neurite outgrowth in SHY-SY5Y and PC12 cells^11^,^12^. Various reports have indicated that the organophosphate (OP) pesticide such as Chlorpyrifos has a critical role in inhibiting the survival of basal forebrain cholinergic neurons in rats via the inhibition of TrkA^13^,^14^. In the last couple of decades, there have been extensive efforts to unravel the mechanistic understanding of impaired neurogenesis and associated disorders due to exposure to environmental chemicals. However, the basic mechanism(s) involved in environmental contaminant induced neurotoxicity are still poorly understood. Organophosphate (OP) pesticides are extremely poisonous to the developing as well as the adult brain^15^. Monocrotophos (MCP), a widely used organophosphate pesticide, is known to be neurotoxic. Thus, we attempted to investigate the role of TrkA activation and its downstream signaling pathways in MCP induced neurotoxicity in cultured rat brain neural stem cell (rBNSCs) derived neuronal cells and rat brain.

Neural stem cells (NSCs) are defined by their ability to self renew, proliferate and differentiate into neurons and glial cells, which are tightly regulated by extrinsic and intrinsic factors. New born neurons and glial cells then migrate to suitable regions in the brain, and incorporate into neural networks for the development of the nervous system. They are not only found in developing brain, but also in different areas of the adult brain and known to be involved in normal brain functioning, including the learning and memory processes, as well as recovery from brain injury^1^,^16^,^17^. NSCs in developing as well as adult brain are vulnerable to environmental neurotoxicants since they adversely affect the brain. Thus, we systematically investigated on primary cultures of rat brain neural stem cells (rBNSCs) and the findings were further correlated with *in-vivo* studies detailed in rat brain following experimental exposure to MCP.

## Results

### *In-vitro* studies

#### Characterization and neural differentiation of rBNSCs

**Figure 1:**
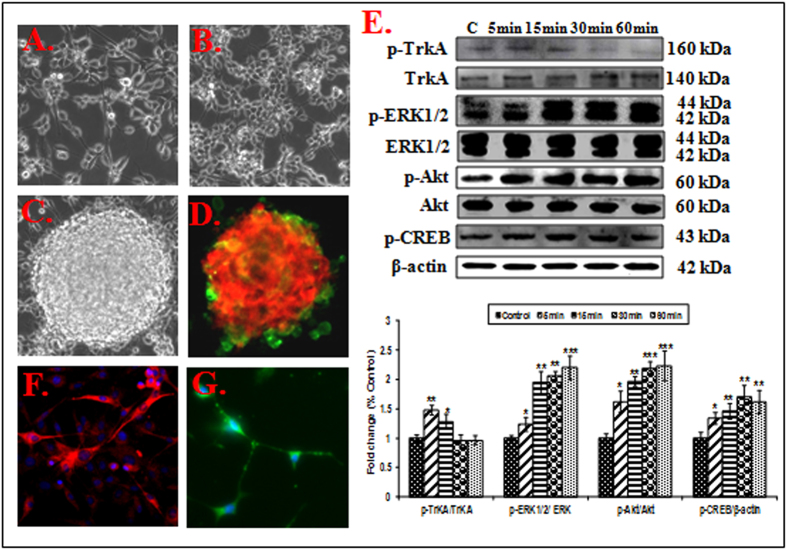
Culture of NSCs: photomicrographs (A–C) represent NSC cultures *in vitro*. NSCs cultured from the embryonic day-12 (ED-12) rat fetuses in presence of mitogens (EGF & bFGF), formed neurospheres. Neurospheres showed immunoreactivity for neural stem and progenitor cell markers nestin (green) and proliferating cells marker BrdU (red) (**D**). TrkA and its downstream molecules (ERK1/2, Akt & CREB) activation by NGF (100 ng/mL) for 5–60 min (**E**). Differentiated NSCs showed neural morphology (**F,G**) and stained positive for cholinergic specific marker ChAT (red) and neural marker NF-M (green) and counter stained with DAPI.

Rat BNSCs isolated from embryonic day-12 (ED-12) rat fetuses showed over 95% viability (Fig. 1A). The proliferative cells were able to develop the small neurospheres by day 7 in serum free neurobasal medium supplemented with growth factors (Fig. 1B). The neurospheres attained maturity by day 20 (Fig. 1C). Cells in neurospheres showed expression of both progenitor cell marker-nestin (green) and proliferating cell marker-BrdU (red) (Fig. 1D). On the exposure to NGF (100 ng/ml) for 5 to 60 minutes, an induction in the phosphorylation of TrkA and downstream molecules (ERK1/2, Akt and CREB) was observed. This induction was maximum, when cells were exposed to NGF for 30 minutes (Fig. 1E). Morphologically differentiated neural cells (over 95%) showed TrkA expression (Supplementary Figure-S1) and cholinergic specific marker ChAT (red color) (Fig. 1F) and neural marker NF-M (green color) (Fig. 1G).

#### MCP inhibits TrkA and its downstream signaling molecules and causes neuronal damage and apoptosis in rBNSC derived neuronal cells

**Figure 2:**
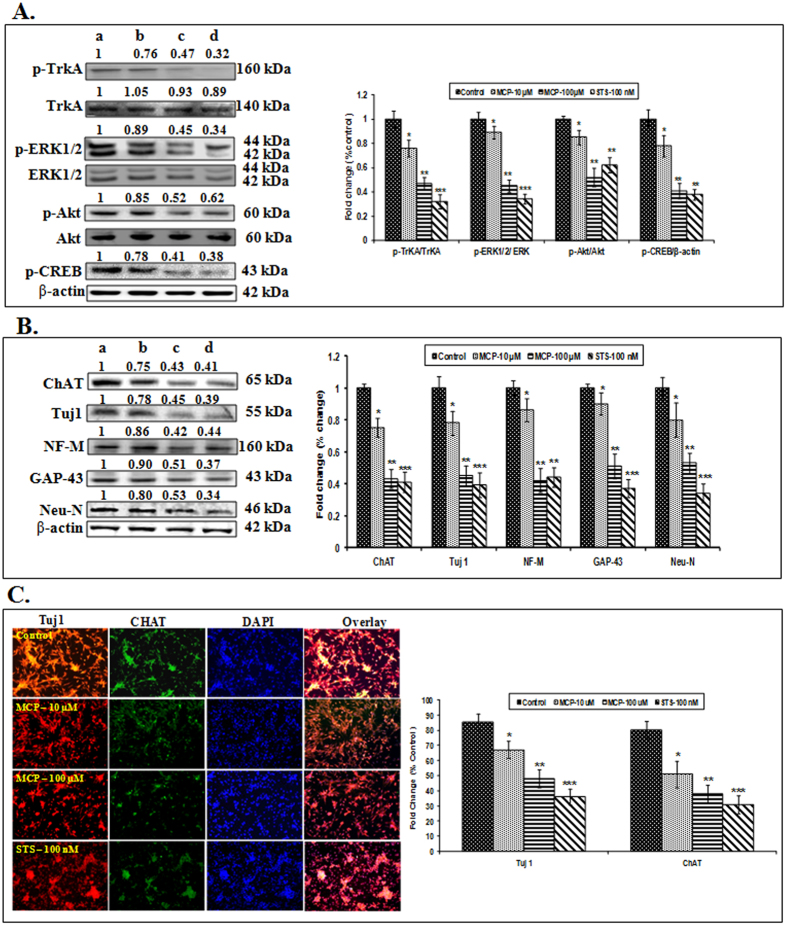
(**A**) Protein expression profiling of TrkA and its downstream molecules in stem cell derived neural cells following the exposures of MCP (10, 100 μM) and STS (100 nM) for 15 minutes. β- actin was used as an internal control to normalize the data. (**a**) Control, (**b**) MCP − 10 μM, (**c**) MCP − 100 μM & (**d**) STS− 100 nM respectively. Molecular weight of protein studied: p-TrkA (160 kDa), TrkA (140 kDa), p-ERK1/2 (44 & 42 kDa), ERK1/2 (44 & 42 kDa), p-Akt (60 kDa), Akt (60 kDa) pCREB (43 kDa) and β- actin (42 kDa) for normalization. Quantification was done in Gel Documentation System (Alpha Innotech, USA) with the help of AlphaEaseTM FC Stand-Alone V.4.0 software. (**B**) Protein expression profiling of neural markers (Tuj1, ChAT, NF-M, GAP43 and NeuN) proteins in stem cell derived neural cells following the exposure of MCP (10, 100 μM) and STS (100 nM) for 24 h. β -actin was used as an internal control to normalize the data. (**a**) Control, (**b**) MCP − 10 μM, (**c**) MCP − 100 μM & (**d**) STS − 100 nM respectively. Molecular weight of protein studied: Tuj1 (55 kDa), NF-M (160 kDa), GAP43 (43 kDa), NeuN (45 kDa), ChAT (65 kD and β - actin (42 kDa) for normalization. Quantification was done in Gel Documentation System (Alpha Innotech, USA) with the help of AlphaEaseTM FC Stand-Alone V.4.0 software. (**C**) Representative microphotographs showing immunocytochemistry localization of neural markers of protein viz. Tuj1 and ChAT in stem cell derived neural cells following the exposure of MCP (10, 100 μM) and STS (100 nM) for 24 h. The images were snapped by Nikon DS-Ri1 (12.7 megapixel) camera. Nuclei stained with DAPI.

**Figure 3:**
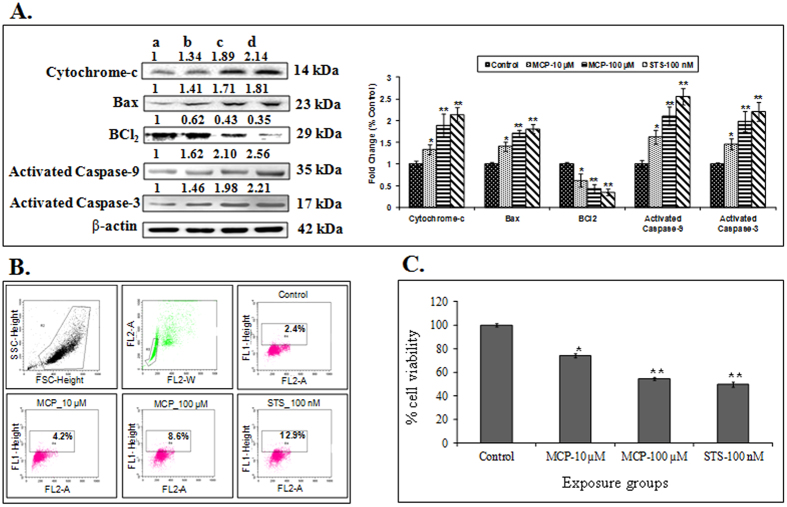
(**A**) Protein expression profiling of marker genes associated with apoptosis in stem cell derived neural cells following the exposures of MCP (10,100 μM) and STS (100 nM) for 24 h. β-actin was used as an internal control to normalize the data. (**a**) Control, (**b**) MCP −10 μM, (**c**) MCP −100 μM & (**d**) STS −100 nM respectively. Molecular weight of protein studied: Cytochrome-C (14 kDa) Bax (23 kDa), BCl_2_ (29 kDa), activated caspase-9 (35 kDa), activated caspase-3 (17 kDa), PARP (116 & 89 kDa) and β- actin (42 kDa) for normalization. Quantification was done in Gel Documentation System (Alpha Innotech, USA) with the help of AlphaEaseTM FC Stand-Alone V.4.0 software. **(B)** DNA damage analysis in stem cell derived neural cells using APO-BrdU™ TUNEL (deoxynucleotide transferase dUTP nick end labeling) Assay Kit with Alexa Fluor® anti-BrdU (Molecular Probes, Invitrogen detection Technologies, USA, Cat No.# A23210) by a flowcytometer (BD-FACS Canto, USA) equipped with BD FACS Diva, version 6.1.2, software. Debris was excluded by forward and side-way light-scattering. (**a**) Control cells, b. MCP-10 μM, c. MCP-100 μM, d. STS-100 nM. **(C)** Cytotoxicity assessed by MTT assay in stem cell derived neural cells following the exposure of MCP (10,100 μM) and STS (100 nM) for 24 h. *p < 0.05; **p < 0.01.

The cells exposed to MCP for 15 minutes inhibited the phosphorylation of TrkA, a signaling molecule known to trigger the cell growth and survival pathways by binding with NGF. The cells exposed to positive control-STS, an inhibitor of TrkA (non-specific), showed a higher magnitude of inhibition of phosphorylation of TrkA. No significant changes were observed in non-phospho TrkA expression when compared with unexposed control (Fig. 2A). The MCP exposure for 15 minutes could not induce any significant change in the protein expression of JNK and p75^NTR^ (data not shown). The other downstream key signaling molecules (Akt, ERK1/2 and CREB), involved in TrkA pathway were also observed to be the significant inhibition in phosphorylation following MCP exposure (100 μM). The lower concentration (10 μM) of MCP was not as effective as to that of higher concentration of MCP (100 μM) and positive control-STS. Such MCP exposure (24 h) induced alterations in the expression of TrkA and its downstream pathway molecules were found to be associated with inhibited expressions of neural markers (Tuj1, NF-M, GAP43, Neu-N) and the expression of cholinergic marker (ChAT) (Fig. 2B & C). The increased expression of cytochrome-c, Bax, caspase-3/9 and reduced expression of BCl_2_ also confirm the MCP induced apoptosis (Fig. 3A). A significant DNA damage (TUNEL assay) in the cells also supports the apoptotic changes (Fig. 3B). All such apoptotic changes were also found to be associated with the elevated levels of reactive oxygen species, malondialdehyde and decreased levels of intracellular glutathione and loss of MMP (Supplementary Figure-S2). A significant loss in cell viability could be associated with these apoptotic changes in the cells exposed to MCP (Fig. 3C).

#### Pharmacological inhibitor studies

**Figure 4:**
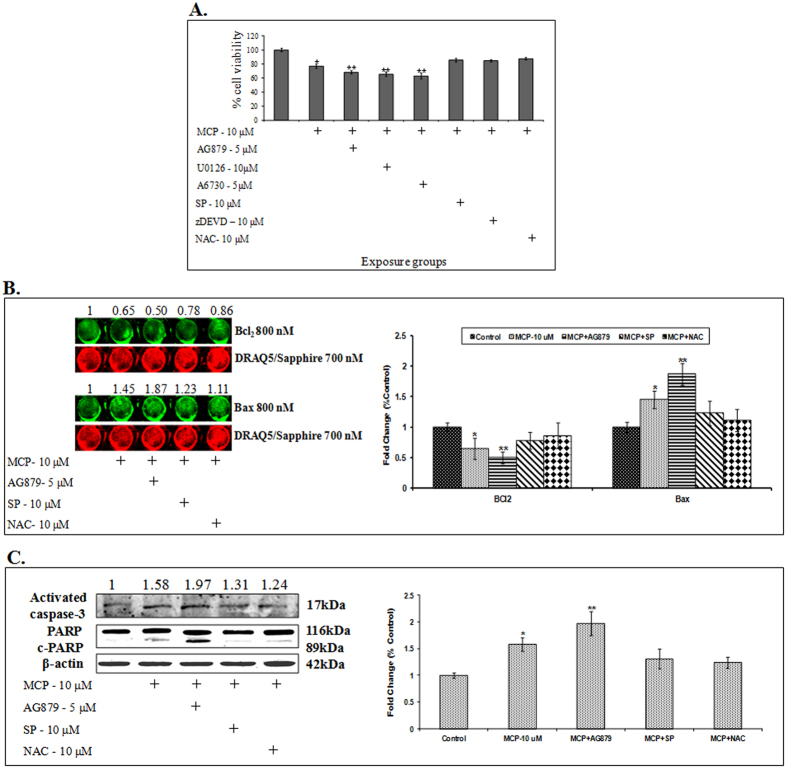
(**A**) Percentage cell viability in cells exposed to MCP (10 μM), or following pharmacological inhibitors against to: TrkA (AG879: 5 μM), MEK1/2 (U0126: 10 μM), Akt (A6730: 5 μM), JNK (SP600125: 10 μM), apoptosis (zDEVD: 10 μM) and anti-oxidant (NAC-10 μM) were introduced 1 h before MCP (10 μM) treatment further 24 h. Data represent means ± SE of four independent experiments. *p < 0.05; **p < 0.01; ***p < 0.001 (unexposed control vs experimental group). **(B)** Expression of caspase 3 and PARP through western Blotting. β-actin was used as a loading control. Cells were exposed to MCP (10 μM), or following inhibitors TrkA (AG879: 5 μM), JNK (SP600125: 10 μM) and anti-oxidant (NAC-10 μM) for 1 h followed by MCP (10 μM) treatment for 24. **(C)** Expression of BCl_2_ and Bax in cells through ICW. Cells were exposed to MCP (10 μM) or following inhibitors TrkA (AG879: 5 μM), JNK (SP600125: 10 μM) and anti-oxidant (NAC-10 μM)for 1 h followed by MCP (10 μM) exposure for 24. Protein changes expressed in relative fold change by comparing the data with respective unexposed control at 800 nM. Sapphire700 stain and DRAQ5 stain was used as normalisation dye at 700 nM. Proteins expressions were analyzed with Odyssey CLX Li-cor software. The data represent means ± SE of four independent experiments. *p < 0.05; **p < 0.01 (unexposed control vs experimental group).

The role of TrkA and its downstream pathway molecules has been confirmed using molecule specific pharmacological inhibitors. Prior to receiving exposure to MCP (10 μM) for 24 h, cells were exposed to pharmacological inhibitors of TrkA (AG879: 5 μM), MEK1/2 (U0126: 10 μM), Akt (A6730: 5 μM), JNK (SP600125: 10 μM) and anti-oxidant (NAC: 10 μM) for 1 h respectively. MCP induced effects became more pronounced in the cells pretreated with specific inhibitors of TrkA (AG879), MEK1/2 (U0126) and Akt (A6730), while specific inhibitors of JNK (SP600125), apoptosis (zDEVD) and anti-oxidant (NAC) have shown promoting effect on cell viability, when the data were compared with the MCP alone exposure (Fig. 4A). Further, ICW analysis was done to study the association of MCP induced loss of cell viability with TrkA signaling mediated apoptosis. TrkA specific inhibitor (AG879: 5 μM) showed significant alterations in the expression of pro- and anti-apoptotic marker proteins (Bcl_2_ and Bax), when compared with the expression of these apoptotic proteins in both MCP exposed and unexposed cells. A reversible response to the MCP induced effects could be recorded in the cells receiving the exposure of specific inhibitors of JNK (SP600125: 10 μM) and NAC (Fig. 4B). In concurrence with these observations, the pre-treatment with a TrkA specific inhibitor (AG879: 5 μM) was found to influence the MCP induced alterations in the protein expression of activated caspase-3, an executer caspase in mitochondria mediated apoptosis. A significant increase in the cleavage of PARP (116 kDa) into 89 kDa fragment was also recorded in the cells pre-exposed to the specific inhibitor of TrkA, when compared with cells exposed to MCP alone (Fig. 4C).

#### Transfection studies

**Figure 5:**
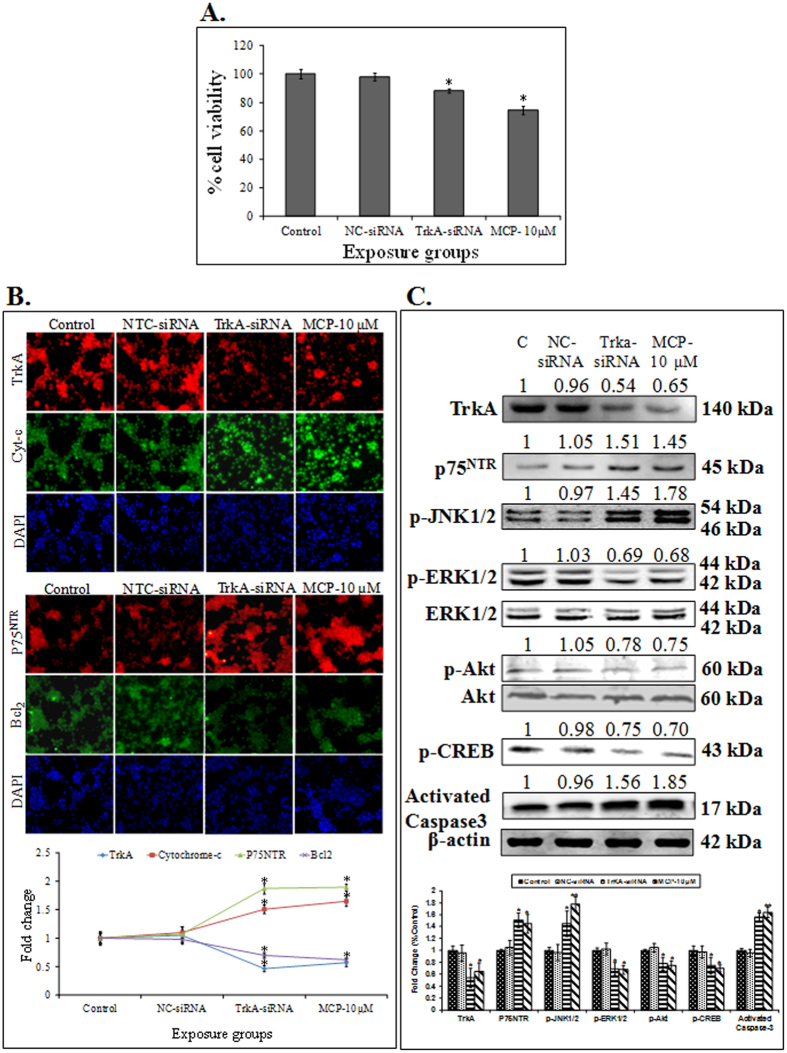
(**A**) Percentage cell viability in specific TrkA si-RNA transfected cells or MCP (10 μM) exposed cells and compared with the basal (unexposed cells) and negative control (only siRNA). The data represent means ± SE of four independent experiments. *p < 0.05; **p < 0.01; ***p < 0.001 (unexposed control vs experimental group). (**B**) Representative microphotographs showing immunocytochemistry localization of protein viz. TrkA, cytochrome-c or p75^NTR^, Bcl_2_ in specific TrkA si-RNA transfected cells, MCP (10 μM) exposed cells or the basal (unexposed cells) and negative control (only siRNA). The images were snapped by Nikon DS-Ri1 (12.7 megapixel) camera. Nuclei stained with DAPI. **(C)** Protein expression profiling of marker proteins of signaling cascades (TrkA, p75^NTR^, p-Akt, Akt, p-ERK1/2, ERK1/2, p-CREB and p-JNK) and apoptosis cells markers (Caspase-3) were studied in specific TrkA si-RNA transfected cells, MCP (10 μM) exposed cells or the basal (unexposed cells) and negative control (only siRNA). β-actin was used as an internal control to normalize the data. Molecular weight of protein studied: Trka (140 kDa), p75^NTR^ (45 kDa), p-Akt (60 kDa),Akt (60 kDa), p-ERK1/2 (44&42 kDa), ERK1/2 (44&42 kDa), pCREB (43 kDa), pJNK1/2 (54&46kDa), activated caspase-3 (17kDa) and β- actin (42 kDa) for normalization. Quantification was done in Gel Documentation System (Alpha Innotech, USA) with the help of AlphaEaseTM FC Stand-Alone V.4.0 software.

We explored the relationship between MCP-induced apoptotic signal transduction and TrkA receptor using RNA interference against TrkA receptor. Cells were transfected with specific TrkA si-RNA and then compared with the cells exposed to only MCP. The data were also compared with the basal (unexposed cells) and negative control (only siRNA). The data show a significant loss in viability in the cells transfected with TrkA-siRNA when compared with cells transfected with non-specific siRNA. The magnitude of viability loss was maximum in the cells exposed to only MCP (Fig. 5A). MCP induced apoptosis via inhibition of TrkA was confirmed in transfected cells by the altered expression of proteins (immunocytochemical analysis) of Trk neurotrophin receptor - p75^NTR^ (with apoptosis marker cytochrome-c) and TrkA (with anti-apoptotic marker Bcl_2_) (Fig. 5B). The increased levels of p75^NTR^ and decreased expression TrkA protein were observed in cells transfected with TrkA-siRNA as well as in those exposed to only MCP, when compared with cells transfected with non-specific siRNA and non-transfected cells. Akt and ERK1/2 pathways are the most prominent downstream cell survival pathways of TrkA, so in the absence of TrkA receptor, p75^NTR^ receptors could mediate a different signaling pathway leading to apoptotic cell death via activation of c-Jun amino- terminal kinase (JNK). For exploring this possibility in our cell system, altered expressions of Akt, ERK1/2, pCREB, and pJNK1/2 proteins were compared between cells transfected with siRNA specific to TrkA receptor and cells exposed to MCP. Cells transfected with non-specific siRNA were used as negative controls while unexposed cells were used as basal controls. Cells transfected with TrkA receptor specific siRNA show a significant down-regulation in the protein expression of pERK1/2, pAkt, and pCREB, while there was an upregulation of activated caspase-3 and pJNK. More or less similar findings were recorded with cells exposed to only MCP. The cells transfected with non-specific siRNA were observed to be expressed near to the basal level (Fig. 5C).

### *In-silico* studies

#### Molecular docking studies-MCP inhibits TrkA receptor activation

Molecular docking studies were carried out to probe whether the MCP induced cell apoptosis was mediated through the inhibition of TrkA, and to gain an insight about the probable nature of MCP binding at the kinase domain of TrkA using crystal structure data. The binding site analysis of co-crystallized ligand AG-23(PDB ID: 4AOJ) revealed that the pyrazole group of the molecule interacts with the residues Glu-590 and Met-592 through H-bond interaction, whereas the isopropoxy group occupied the space between the residues Phe-589, Leu-567 and Ala-542^18^. The pyrimidine N3 atom binds to Asp-596 NH through a water molecule in the solvent-exposed region of the ATP binding site. The fluoropyridine ring of the AG-23 was placed in the hydrophobic cavity formed by the amino acid residues Leu-657, Asn-665, and Gly-667.

**Figure 6:**
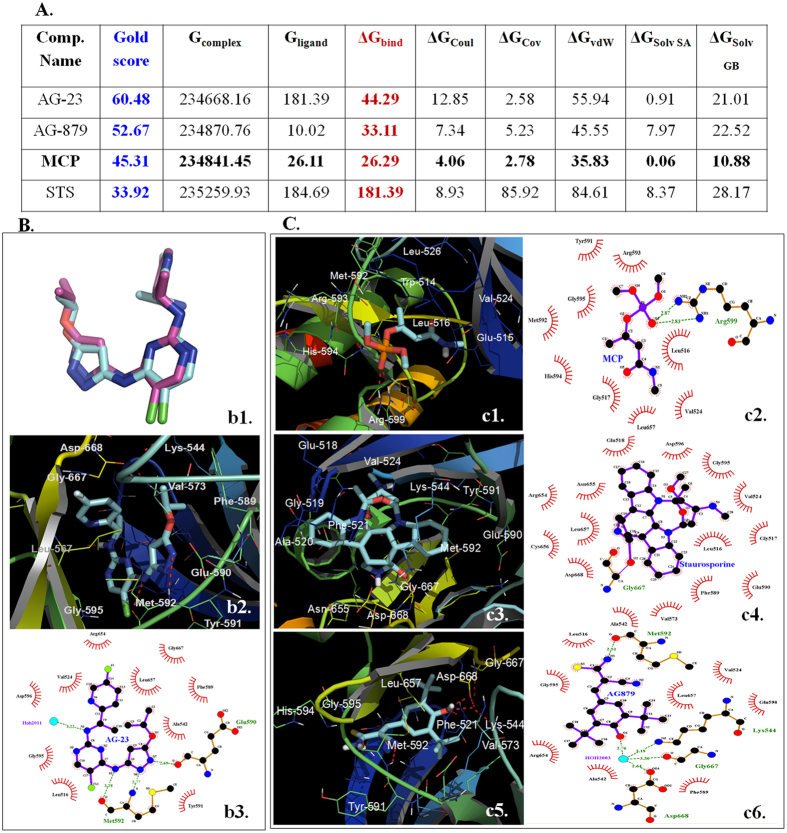
(**A**) Summary of the MM-GBSA end-point free energies for the TrkA inhibitors. **(B)** Comparison of conformation for the AG-23 in the co-crystallized (pink colored carbon) and docked (cyan colored carbon) binding pose of TrkA. **(C)** Comparative 3D and 2D binding pose view of MCP **(c1** and **c2)**, STS (**c3** and **c4)**, and AG-879 (**c5** and **c6)** at the active site of TrkA.

The co-crystallized inactive form of human TrkA protein (PDB ID: 4AOJ) with the inhibitor AG-23 was chosen for the molecular docking studies, and two non-specific inhibitors of tyrosine kinases (MCP and STS) as well as known TrkA inhibitor AG879 were subjected to rigorous docking and scoring protocol. To evaluate the correct binding prediction of the docking program, the co-crystallized ligand AG-23 was initially docked at the active site of the human TrkA protein. The comparative analysis of binding site between docked poses and X-ray crystal structure of AG-23 revealed that there is no significant change in the binding mode of AG-23, both in terms of ligand conformation and the binding amino acid residues, thus suggesting the correct binding prediction of docking program (Fig. 6B).

Figure 6A summarizes the GOLD docking score along with the free energies (ΔG) for each protein-ligand complex. Among the four TrkA inhibitor molecules, AG-23 showed highest docking score, while the docking score of MCP was less than AG-879. We extended the studies to calculate the free energy (ΔG) for each protein-ligand complex, which gave more insights about the inhibitor binding. The binding free energy (ΔG_bind_) data correctly corroborated with the experimental TrkA inhibitory activity, where the binding free energy (ΔG_bind_) for STS was maximum (181.39 kcal/mol) among the four TrkA inhibitors, followed by AG-23, AG-879 and MCP. The least docking score as well as the value of ΔG_bind_ of MCP indicated that although MCP inhibits the TrkA activity, its inhibitory potency is weak among the four TrkA inhibitors given in Fig. 6A.

The binding pose analysis of MCP at the kinase domain TrkA revealed that it’s double bonded oxygen of the phosphate group showed the H-bond interaction with Arg599 residue, while the methyl group was attached with O-4 atom and placed towards the cavity surrounded by the residues Arg593, Tyr591, and Gly595. The methylamino group occupied the cavity surrounded by the residues Leu516, Val524, Leu657 and Gly517, while the methyl group attached to C-2 carbon was directed towards Met592 and Arg593 (Fig. 6C).

The docking pose analysis of STS at the kinase domain TrkA revealed that its methoxy, amino and methyl groups at one end were occupied by solvent-exposed region surrounded by the residues Leu-516, Gly-517, Val-524, Gly-595 and Asp-596, whereas, at the other end the ketonic group was joined by indole nucleus exposed to solvent-accessible residues namely Leu-657, Asp-668, Ala-542 and Val-573. The ketonic oxygen of STS showed the covalent interaction with Gly-667 resulting in its highest inhibitory potency. The docking pose analysis of AG-879 at the kinase domain TrkA revealed that the amino end of the molecule was involved in H-bond interaction with crucial Met592, while the hydroxyl moiety attached to the phenyl group showed water mediated H-bond interaction with the residues Asp-668, Gly-667 and Lys-544. The tert-butyl group attached with C9 atom of the molecule was exposed to the cavity formed by the residues Gly-667, Lys-544, Leu-657 and Val-573, while the tert-butyl group attached with C7 atom of the molecule was surrounded by the residues Ala-542, Arg-654 and Gly-595.

### *In Vivo* studies

#### MCP induces apoptosis in rat brain by altering the TrkA and p75^NTR^ mediated signaling

**Figure 7:**
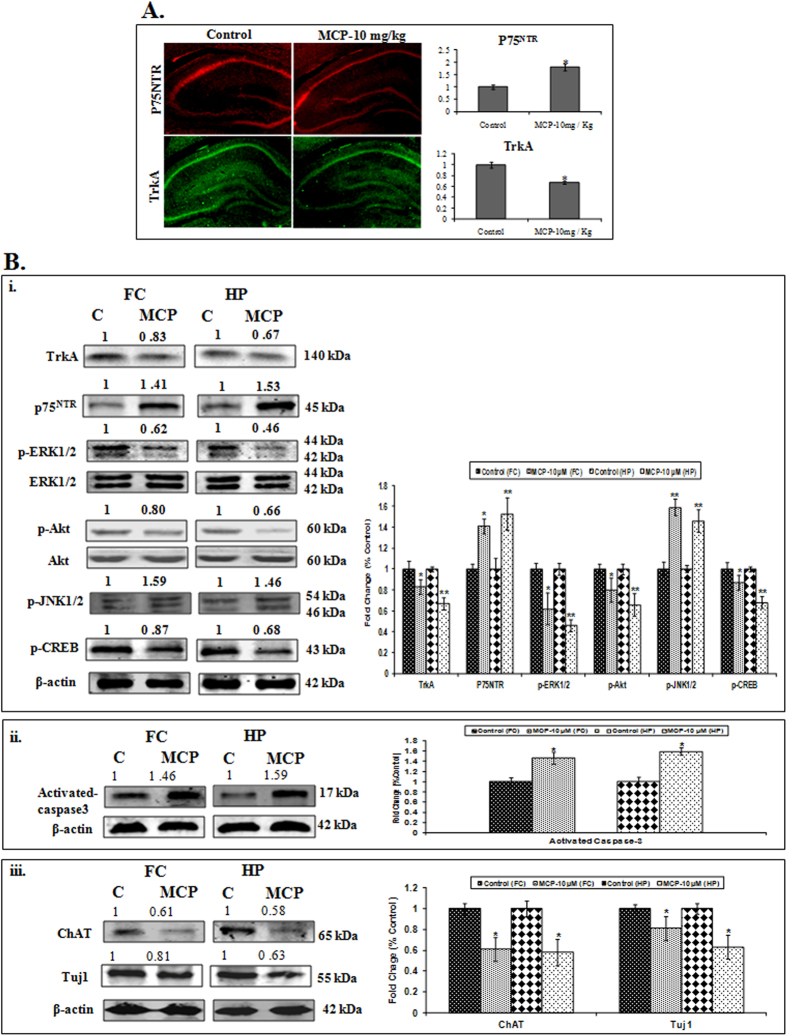
(**A**) Immunohistochemical analysis to determine expression level of TrkA and p75^NTR^ in the hippocampus after treatment with MCP-10mg/Kg Body weight. (**B**) (**i**). Protein expression profiling of marker proteins of signaling cascades vitz. TrkA, p75^NTR^, p-Akt, Akt, p-ERK1/2, ERK1/2, p-CREB and p-JNK, (**ii**). apoptosis cells marker caspases-3 and (**iii**). neural markers such as Tuj1 & ChAT and were studied in the frontal cortex and hippocampus regions of rat brain following the exposures to MCP (10 mg/kg body weight, p.o., for 7 days). β -actin was used as an internal control to normalize the data. Molecular weight of protein studied: TrkA (140kDa), p75^NTR^ (45kDa), p-Akt (60kDa), Akt (60kDa), p-ERK1/2 (44 & 42kDa), ERK1/2 (44 & 42kDa), pCREB (43kDa), pJNK1/2 (54 & 46kDa) Tuj1 (55kDa), ChAT (65kD), activated caspase-3 (17kDa) and β- actin (42kDa) for normalization. Quantification was done in Gel Documentation System (Alpha Innotech, USA) with the help of AlphaEaseTM FC StandAlone V. 4.0 software.

The protein expression studies were carried out for TrkA/p75^NTR^ system, Akt/ ERK signaling pathway molecules and JNK pathways for apoptosis signaling in the brain of adult rats exposed to MCP (10 mg/kg body weight, p.o., single dose). Both immuno-histochemistry and western blot data showed a significant inhibition in the TrkA activation and up-regulation of p75^NTR^ in the hippocampus of the brain of animals exposed to MCP. Although, MCP induced downregulation in the expression of TrkA and increased the levels of p75^NTR^ were observed in both hippocampus and frontal cortex, the effect was more prominent in hippocampus region (Fig. 7A). In concurrence with the *in-vitro* findings, we observe that MCP exposure significantly down-regulates the expression of pERK1/2, pAkt, and pCREB, while up-regulates the expression of pJNK in both frontal cortex and hippocampus regions of the brain (Fig. 7B i). The magnitude of altered expression of these proteins was higher in hippocampus than in frontal cortex region. The subsequent effect in the expression of marker proteins of apoptosis caspases 3 and neuronal markers (Tuj1, ChAT) was also recorded in both hippocampus and frontal cortex regions of exposed brain (Fig. 7B ii & iii). MCP induced activation of these apoptotic cascades have also led to significant DNA fragmentation which was confirmed by TUNEL assay (Supplementary Figure-S3).

#### Neurobehavioral studies

**Figure 8:**
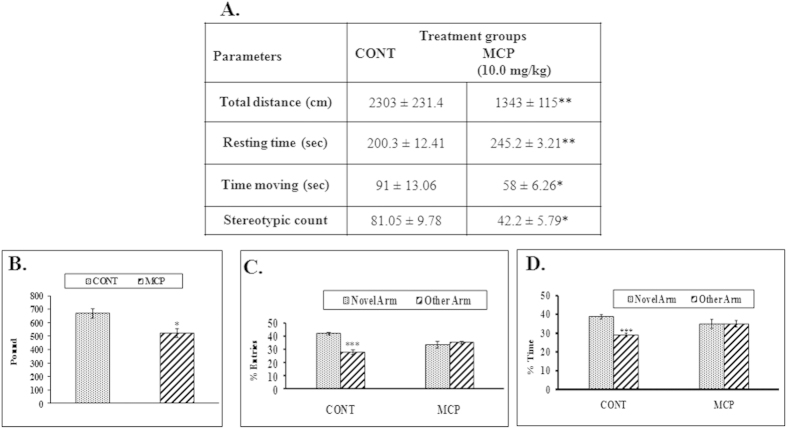
(**A**) Effect on motor activity following exposure of rats to MCP for 7 days. Values are mean ± SEM of five animals in each group. Significantly differs (*p < 0.05, **p < 0.01) as compared to control. (**B**) Effect on fore-limb grip strength following the exposure of rats to MCP for 7 days. Values are mean ± SEM of five animals in each group. Significantly differs (*p < 0.05) as compared to control. (**C**) Effect on spatial learning and memory based upon percentage of entries following exposure of rats to MCP for 7 days. Values are mean ± SEM of five animals in each group. Significantly differs (***p < 0.001) compared to novel arm versus other arm. (**D**) Effect on spatial learning and memory based upon time spent following exposure of rats to MCP for 7 days. Values are mean ± SEM of five animals in each group. Significantly differs (***p < 0.001) compared to novel arm versus other arm.

MCP exposed rats showed a significant impairment in spontaneous locomotor activity as reflected by the decrease in total distance travelled (41%, p < 0.01), moving time (36%, p < 0.05), stereotypic count (47%, p < 0.05) and increase in resting time (22%, p < 0.01) as compared to the control group (Fig. 8A). A significant (21%, p < 0.05) loss of forelimb grip strength was also recorded in MCP exposed rats (Fig. 8B). MCP exposure also induced significant (p < 0.001) deficits in learning and memory in the experimental rats as confirmed by ‘Y’ maze analysis (Fig. 8C & D). The neurobehavioral studies confirmed significant functional impairment in spontaneous motor activity, grip strength and learning memory in rats experimentally exposed to MCP.

## Discussion

The multiple effects of TrkA including NGF-induced neuronal differentiation and hindrance of programmed cell death has been widely studied^8^,^19^,^20^. NGF is known to play a key role in the development of neuronal cells of central as well as peripheral nervous systems^21^. NGF and other neurotrophins bind to specific receptors, tyrosine kinases (Trks)/p75^NTR^ at the cell surface and activate them for differentiation/ apoptosis. Such events also initiate the internalization of the activated receptors into vesicles, hence remain active for a longer period^22^. The binding of NGF to its specific receptors viz., TrkA and p75^NTR^ leads to a series of downstream signaling events which ultimately mediates neuronal survival, differentiation, and maintenance. PI3K/Akt/β-Catenin and MAPKs are major pathways involved in neuronal survival and differentiation^4^,^23^. Though, we have earlier reported MCP-induced impairment in survival and differentiation in rat pheochromocytoma cells by altering these pathways^24^,^25^ but the role of TrkA/p75^NTR^ signalling on these alterations have not been determined so far. Thus, we investigated the effects of MCP on regulating TrkA/p75^NTR^ trafficking, associated downstream signaling events and subsequent injuries in neuronal cells derived from rat brain neural stem cells (rBNSCs).

It has been demonstrated that TrkA signaling plays a key role in the induction of proliferation and differentiation of neural stem cells (NSCs) during brain development^3^,^26^, and altered levels of TrkA receptor have been found to be associated with neurodegeneration and disease progression in Alzheimer’s and Parkinson’s disease^27^,^28^. Biological interactions between environmental toxicants and brain NSCs may lead to irreparable damages in the brain^29^,^30^. Hence, NSCs could be an adequate system of choice for studying chemical induced neural injury and repair. In the present investigation, we show that the neuronal cells derived from rat brain neural stem cell (rBNSC) have the significant activity of TrkA and its downstream molecules ERK1/2, Akt and CREB and a range of phosphorylation by NGF after the withdrawal of mitogenic factors i.e., EGF and bFGF. These neuronal cells were having the significant expression of neuronal markers including cholinergic ChAT and neuronal morphology.

We investigated if the MCP induced apoptosis and subsequent neurotoxicity in neuronal cells derived from rBNSC was triggered through TrkA signaling. Our results demonstrate a significant inhibition in the phosphorylation of TrkA and subsequent inhibition in the TrkA mediated downstream key signaling molecules that lead to inhibition of pro-survival ERK1/2 and Akt signaling in stem cell derived neuronal cells, exposed to MCP. Such MCP induced inhibition in the expression of TrkA and other signaling molecules was found to be associated with the altered expressions of neural markers (Tuj1, NF-M, GAP43, Neu-N) including cholinergic (ChAT) expression. The increased expression of cytochrome-c, Bax, caspase-3/9 and reduced expression of BCl_2_ in our studies have also confirmed the mitochondria mediated caspase cascade induced apoptosis due to MCP exposure. Further, a significant DNA damage, oxidative stress and subsequent loss of cell viability also supports the MCP induced apoptotic changes.

The MCP induced inhibition in the pathways of survival signals and activation of death signals was further confirmed using pharmacological inhibitors. We observed that cells pre-treated with JNK1/2 inhibitor (SP600125) protect themselves from MCP-induced apoptosis, which suggests the critical involvement of the JNK pathway in MCP mediated toxicity in neural cells derived from rBNSCs. Similarly, pre-treatment of inhibitors of TrkA, ERK1/2 and Akt significantly increased the MCP induced cell death in comparison to the experimental group exposed to MCP alone. These findings also suggest the critical role of activation of TrkA, ERK1/2 and Akt in anti-apoptotic activity against MCP exposure. Our data also correlate the TrkA mediated ERK1/2 and Akt inhibition with MCP induced apoptosis and neuronal injuries. The data show that MCP-induced cell death involves not only inhibition of survival signals, but also simultaneous activation of a death signal by p75^NTR^. The inhibition of TrkA expression is known to activate the p75^NTR^ mediated JNK1/2 activity^31^. We also got a simultaneous activation of p75^NTR^ in MCP exposed cells, lead apoptosis through the p75^NTR^ mediated JNK1/2 activation. The p75^NTR^ has a complex role in regulating neural survival and death that is dependent on its binding to ligands and co-receptors^32^. The p75^NTR^ exerted mechanisms of inhibition of TrkA have been suggested as one among the important factors accelerating neurodegenerative disorders^33^,^34^. Thus, to find out the cross talk between TrkA receptor inactivation and p75^NTR^ receptor activation, we made a TrkA receptor gene expression silencing using specific siRNA in rBNSCs. The substantial reduction in the expression of TrkA was recorded in TrkA specific siRNA transfected cells, which was comparable to the reduced expressions of TrkA due to MCP exposure in the other set of cells. The cells transfected with non-specific siRNA did not show any reduction in the expression of TrkA. These reduced expressions of TrkA were inversely proportional to the increased expression of p75^NTR^ in TrkA specific siRNA transfected and MCP exposed cells. The findings demonstrated the triggering of MCP induced neurotoxicity by inhibition of TrkA signaling and activation of p75^NTR^ cascades. The decrease in the TrkA expression and a parallel increase in the level of p75^NTR^ has also been reported during normal aging as well as in AD^35^,^36^ and suggests that such individuals may be more susceptible to organophosphate pesticide related neurotoxicity.

The involvement of TrkA/p75^NTR^ dependent signaling cascades including ERK1/2, Akt and JNK1/2 signaling pathway in MCP induced neurotoxicity was reconfirmed by significant downregulated expression of pERK1/2, pAkt, pCREB along with upregulation of activated pJNK. These events could have been correlated well with altered markers of apoptosis signaling and decreased expression of cell survival pathways, thus suggesting neurodegeneration. Since, a fine balance between ERK1/2, Akt and JNK1/2 signaling pathways has been demonstrated as an important event in the maintenance of proper neural cell proliferation and differentiation during brain development^37^,^38^. ERK1/2 upon activation by TrkA leads to increase in the expression of transcription factor CREB^39^ that plays an important role in maintaining neural plasticity, survival and long-term memory^40^, while Akt phosphorylated by TrkA is considered to be one of the keys of pro-survival pathways within the cell and regulate apoptosis^41^. The impairment of Akt signaling activates the mitochondria mediated apoptosis process as confirmed by the increased expressions of cytochrome-c, the executor caspase to induce the apoptotic cell death^42^,^43^.

We are reporting for the first time that MCP mimics TrkA receptor ligand and binds to the ATP binding domain of TrkA receptor, thereby inhibits its activation. The molecular docking studies demonstrate that MCP induced apoptosis was mediated through the inhibition of phosphorylation of TrkA by binding at the kinase domain of TrkA. The binding free energy (ΔG_bind_) data correctly corroborated with the experimental TrkA inhibitory activity where the binding free energy (ΔG_bind_) for MCP was considerably high. The docking pose homology in terms of interaction of MCP, STS and the known TrkA inhibitor-AG879 at the kinase domain of TrkA corroborates the observed TrkA inhibition effect of MCP. The molecular docking studies reveal that the STS and MCP follow a similar binding pattern as with the AG-879 (well known TrkA inhibitor) and co-crystallized at similar position as the ligand of TrkA (PDB id: 4AOJ) at the ATP binding domain. This led to the inhibition of TrkA and resulting cascades. The main cause of MCP mediated TrkA inhibition is considered to be due to water mediated H-bond interaction, and strong interaction with Met-592 similar to the co-crystallized ligand.

Though, the finding of the present investigations relveal the MCP induced neurotoxic reposnses are primarily mediated through TrkA/p75^NTR^ dependent signaling cascades, but our previous findings for MCP also confirm the significant involvement of Akt. These *in silico* studies indicated that MCP has the binding affinity for kinase protein Akt and play a critical role in MCP induced neurotoxicity in stem cell derived neuronal cells of human^44^. Therefore, the docking studies together suggest that the MCP may occupy the common binding site present in both of these proteins resulting into the inhibition of these proteins. Based on the findings of our previous and present investigations, it is also being inferred that MCP has a non-selective inhibitor of Akt and TrkA.

**Figure 9:**
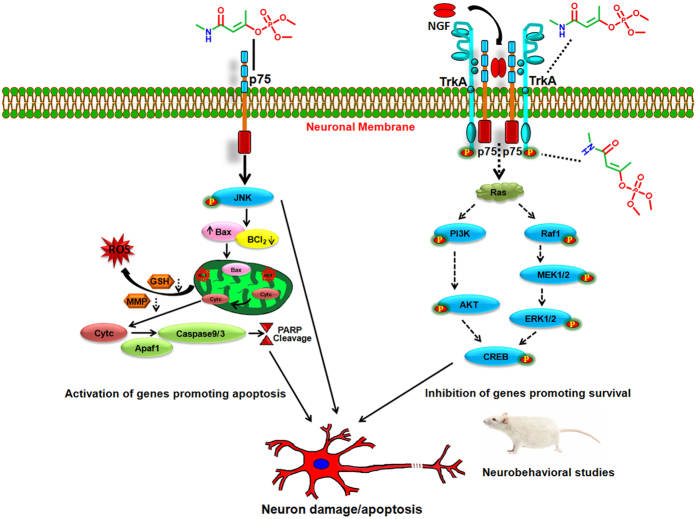
Schematic diagram illustrating the MCP-induced neurodegeneration/apoptosis and neurobehavioral abnormalities/cognitive impairment. The figure is designed and drawn by the author Dr Vivek Kumar and has no copyright material in it. The figure is submitted for the first time to publish in this manuscript only.

In concurrence to the *in-vitro* and *in-silico* findings, the significant neurotoxic responses and activation events associated with apoptotic signaling cascade and DNA damage were observed in hippocampus and frontal cortex regions of the brain of rats exposed to MCP. These neurotoxic responses could be well correlated with standard endpoints of neurobehavioral deficits viz., locomotor activity, grip strength and Y maze, which finally reflect cholinergic impairment^45^,^46^. Similar to *in vitro* findings, there was a significant inhibition in the expression of TrkA and associated downstream pathway molecules, while upregulation of p75^NTR^ signaling molecules was observed in the hippocampus and frontal cortex regions of rats exposed to MCP. These findings together suggest that MCP induces neurotoxicity by altering the molecular mechanism of switching of TrkA/p75^NTR^ signaling. The exposure to MCP could be a potential threat for further neurodegeneration in patients of AD, PD and other neurodegenerative diseases. The schematic diagram showing the possible mechanism of action of MCP induced neural cell death could be drawn by observing the results of the current study (Fig. 9).

## Conclusion

We have demonstrated the molecular switching mechanism of TrkA/p75^NTR^ signaling in MCP induced neurotoxicity under both *in-vitro* (cultured rat brain derived neural cells) and *in-vivo* (rat brain) conditions. Since, both the receptors, i.e., TrkA and p75^NTR^ bind to NGF and known to interact, it may be possible to fine tune for their proper action in the form of proliferation, differentiation and injuries^47^. The data also identify the neurotoxicity mechanisms of organophosphate pesticide-MCP, and provides useful information to evince that pesticide-induced neurotoxicity may be an important risk factor for neurodegenerative diseases. The exposure to this pesticide may further increase the intensity of neurodegeneration in the patients suffering from AD, PD and other neurodegenerative disorders.

## Material and Methods

### Reagents and consumables

All the specified chemicals, reagents, diagnostic kits were purchased from Sigma Chemical Company Pvt. Ltd. St. Louis, MO, USA, unless otherwise stated. Culture medium, antibiotics, serum and growth factors were purchased from Gibco BRL, USA.

### *In Vitro* studies

#### Isolation and characterization of neural stem cells (NSCs)

NSCs were isolated from the embryonic day-12 (ED-12) rat fetuses (elaborated in supplementary material). Small proliferating neurospheres appeared after 1 week, which matured by day 20. The neurosphere culture was passaged at an interval of 10–12 days by gently triturating the neurospheres and re-plating the single cell suspension of NSCs. The NSCs were characterized by using anti-nestin and anti-BrdU antibody (elaborated in supplementary material.

#### Neuronal differentiation of NSCs

After passage # 5, dissociated neurospheres were plated on poly-L Lysine (PLL) coated flasks (5 × 10^4^ cells/Cm^2^) in serum free neurobasal medium containing DMEM: F12 (1:1) supplemented with N-2 (1%), B-27 (2%) and mitogenic factors (10 ng/ml bFGF & EGF). For inducing cholinergic differentiation, cells were incubated in differentiation medium containing NGF (100 ng/ml) with reduced concentrations of EGF and bFGF (1 ng/mL), B-27 (0.1%) and N-2 (1%). Immediate after placing the cells in the differentiation medium, the analysis was done to study the initiation in the expression of signaling cascade molecules involved in neural differentiation i.e., TrkA and its downstream molecules (ERK1/2, Akt and CREB). The expression of signaling molecules was assessed for 5 to 60 min. Differentiation medium was changed every alternate day. Neuronal differentiation was confirmed by assessing the morphological appearance and expression of cholinergic (ChAT) and neurofilament medium (NF-M) proteins. For the experimental purpose, confluent growing cells were sub-cultured in PLL pre-coated six-well culture plate and 75-Cm^2^ culture flask.

#### Identification of non-cytotoxic doses of monocrotophos (MCP)

Prior employing in the expression studies, non-cytotoxic doses of MCP were ascertained using standard endpoint i.e., tetrazolium bromide MTT assay as described by Agrawal *et al.*^48^.

#### Exposure schedule for in vitro studies

Based on the data of cytotoxicity studies (Supplementary Figure-S4), differentiated cells were exposed to MCP (10–100 μM) for 24 h. Unexposed sets were also run under identical conditions and served as basal control. The cells exposed to staurosporine (100 nM) were used as positive control. After exposure, cells were studied for markers associated with apoptosis, signaling pathways involved in apoptosis/neurogenesis.

#### Apoptosis studies

Deoxynuclotide transferase dUTP nick end labeling (TUNEL) assay using APO-BrdU TUNEL Assay Kit with Alexa Fluor 488 anti-BrdU (Molecular Probes, Invitrogen Detection Technologies, USA, Catalog No. A23210).

#### Cell signaling pathway, neural markers and apoptosis marker studies

The altered expression of marker proteins of signaling cascades (P-TrkA, TrkA, p75^NTR^, p-Akt, Akt, p-ERK1/2, ERK1/2 and p-CREB, p-JNK), neural markers (TUJ1, NF-M, Neu-N, GAP43, ChAT) and apoptosis markers (Bax, BCl_2_, Cytochrome-c, Caspases 9/3 and PARP) were studied in stem cell derived neuronal cells. Proteins harvested from experimental and control groups were processed for Western blot analysis following our protocol^49^. The immunocyotochemical localization of the signaling marker proteins was also done following the protocol described earlier^50^,^51^.

#### Signaling molecule inhibition studies

The role of TrkA and its downstream pathway molecules has been confirmed using molecule specific pharmacological inhibitors. The cells were seeded in PLL pre-coated 96 well culture plates and allowed to adhere for 24 h prior to the experimental exposure. The cells were exposed in two different groups i.e., MCP (10 μM) for 24 h (Group-1) and in Group-2, cells were exposed to either of pharmacological inhibitors of TrkA (AG879: 5 μM), MEK1/2 (U0126: 10 μM), Akt (A6730: 5 μM), JNK (SP600125: 10 μM), apoptosis (zDEVD: 10 μM) and anti-oxidant (NAC-10 μM) for 1 h prior the exposure to MCP (10 μM) for 24 h. The cells of both the groups were processed for MTT assay.

#### In-cell western (ICW) assay

ICW was carried out for specific marker proteins i.e., Bax and BCl_2_ using ICW kit of LI-COR Biosciences as described by the manufacturer. Briefly, 10,000 cells were seeded in each well of 96 well plates and exposed to MCP (10 μM) or to the following inhibitors of TrkA (AG879: 5 μM), JNK (SP600125: 10 μM) and anti-oxidant (NAC-10 μM) for 1 h followed by MCP (10 μM) exposure for 24 h. Cells were washed with PBS and fixed with 3.7% paraformaldehyde for 20 minutes. After fixation, cells were washed three times with 1X PBS containing 0.1% triton X-100 for permealization. Blocking was done by incubating in Odyssey blocking buffer for 1.5 h at room temperature. Primary antibody against Bcl2 (1:100) and Bax (1:100) was diluted in blocking buffer and added to the cells and the plates were gently shaked for 3 h. After 3 hours of incubation, plates were washed with PBS containing 0.1% tween-20 thrice. Similarly, secondary antibody raised against rabbit and labeled with IR Dye 800 CW was diluted in Odyssey blocking buffer at 1:800 dilution. The secondary antibody solution also contained a mixture of Sapphire700 stain (1:1000) and DRAQ5 stain (1:10,000), which acts as a normalisation dye. The above mixture was then added to the cells and gently shaken for 1 h at room temperature in dark. Finally, the cells were washed with PBS containing 0.1% tween-20 thrice by gentle shaking. Before scanning on LI-COR, Odyssey CLx machine, plates were dried by inverting them on tissue paper. After imaging at 700 and 800 nm channels, analysis was done using Image Studio 2.1.1.0

#### Western blot analysis

The involvement of TrkA and its downstream signaling molecules was also studied by western blot analysis. The experimental groups and exposure period were identical as were used in in-cell western assay.

#### Transfection study

The involvement of the TrkA mediated signaling cascade was further confirmed by blocking the expression of the TrkA receptor using specific siRNA (Silencer R selected pre-designed SiRNA, Ambion, 4390771) and compared it with the altered expression of TrkA and mediated signals with MCP exposed cells. For studying the specific activity of TrkA receptor siRNA, a set of cells transfected with negative control siRNA (on target plus control pool non-targeting pool, Thermo Scientific, D-001810-10-05) was also run under identical conditions. Unexposed cells were used as basal control. About 80% transfection efficiency was achieved and confirmed (supplementary figure-S5) by CY3 siRNA (silencer CY3 labeled negative control siRNA, Ambion, AM4621)) using Lipofectamine 2000 (Invitrogen). Cells were used in the study post day 2 of transfection. The analysis was done using percent cell viability (MTT assay) and altered protein expression (western blotting and immunocytochemistry).

### *In Silico* studies

#### Molecular docking protocol

The docking experiments were carried out using the GOLD docking program (GOLD, Version 3.1; Cambridge Crystallographic Data Centre: Cambridge, UK). The atomic coordinates of TrkA (PDB ID: 4AOJ) were downloaded from the Brookhaven Protein Data Bank (www.rcsb.org) and prepared using a protein preparation wizard where bond orders were assigned, water and other residues except bound ligands were deleted, and finally the protein was backbone constrained minimized using OPLS-2005 force field implemented in Schrödinger software package (Schrödinger, version 9.1; Schrodinger, LLC: New York, 2005). The bond conformation of co-crystallized AG-23 was used as control for defining the active site in TrkA. The optimized 3D-structures of new ligands were docked within 10 Å radius by running 20 genetic algorithm (GA) steps for each. The docked poses of geometry optimized ligands were ranked using the Gold Score (GS), and were used to find the most optimal binding pose of each ligand. In the GOLD program, the default parameters such as population size (100); selection-pressure (1.1); number of operations (10,000); number of islands (1); niche size (2); and operator weights for migrate (0), mutate (100) and crossover (100) were applied. The top five binding poses of each ligand were analyzed, to select the best binding pose of each ligand, in order to untangle the essential parameters in terms of direct- (H-bonds) and indirect (hydrophobic) interactions governing binding disparities among the series of these compounds. The binding free energies (ΔG bind) for each protein-ligand complex were calculated using Generalized Born Surface Area (MM/GBSA) methods implemented in Schrödinger software package (Schrödinger, version 9.1; Schrodinger, LLC: New York, 2005).

### ***In** Vivo* studies

#### Animals and treatment schedule

The protocol for the study was approved by the Institutional Animal Ethics Committee of CSIR-Indian Institute of Toxicology Research (CSIR-IITR), Lucknow, India, and all experiments have been carried out in accordance with the guidelines laid down by the committee for the purpose of control and supervision of experiments on animals, Ministry of Environment and Forests (Government of India), New Delhi, India. Wistar rats weighing 180 ± 20 gm obtained from the animal breeding colony of CSIR-IITR, Lucknow, India, were housed in polypropylene cages under standard hygiene conditions with a 12 hour light/dark cycle at 25 ± 2 °C and fed a pellet diet (Ashirwad Industries, Chandigarh, India) and water *ad libitum*. Following randomization and acclimatization, animals were divided into experimental and control groups. The single dose of MCP (10 mg/kg body weight) was administered by oral gavage to the experimental animals, while the vehicle control group was given corn oil under identical experimental conditions. MCP induced alterations in the functional phenotypic expression were evaluated by key neuro-behavioral endpoints such as, locomotor activity, grip strength and ‘Y’ maze a day prior to sacrificing the animals i.e., 7^th^ day after the dosing^52^. The other set of animals was used to study regional specific marker proteins in two different parts of the brain. Following perfusion, brain was collected immediately, washed in ice cold normal saline (0.9% NaCl) and dissected into brain regions following the standard procedure of Ansari *et al.*^53^ and processed for immunohistochemistry and western blot analysis.

#### Immunohistochemistry

Immunohistochemical studies were carried out following the method of Ansari *et al.*^53^. The intensity of anti-TrkA and anti-p75^NTR^ in the hippocampal region of the brain was determined using a computerized image analysis system (Leica Qwin 500 image analysis software) as described by Shingo *et al.*^54^.

#### Western blot analysis

After exposure to MCP, expression changes in the frontal cortex and hippocampus region of rat brain was assayed following the method of Yadav *et al.*^55^. The marker proteins analyzed to study the altered expression were same as studied in the *in vitro* studies. The brain regions were homogenized and proteins were isolated for further processing as per the protocol of Yadav *et al.*^55^.

#### Behavioral studies

- **(a) Spontaneous motor activity:** Spontaneous motor activity in rats was assessed using computerized Actimot (TSE, Germany) following the method as described by Yadav *et al.*^56^. Effect on different parameters including total distance travelled, resting time, time moving and stereotypic count was studied in the exposed groups.
- **(b) Grip strength:** A computerized grip strength meter (TSE, Germany) was used to measure the forelimb grip strength in the control and exposed rats following the standard procedure as described by Yadav *et al.*^56^.
- **(c) Y maze:** Spatial memory task was tested by using Y maze (TSE, Germany) following the procedure of Wang *et al.*^57^. Entry to the arms was scored as soon as the front paws of the rats crossed into an arm. The results are expressed in percentage of time spent and percentage of entries in comparison to novel arms versus other arms.

#### Statistical analysis

Results are expressed as mean ± standard error of mean (SEM) for the values obtained from at least three independent experiments. Statistical analysis was performed using one-way analysis of variance (ANOVA) using Graph Pad Prism (Version 5.0) software. The values *p < 0.05 were considered as significant, **p < 0.01 more significant and ***p < 0.001 highly significant.

## Additional Information

**How to cite this article**: Kumar, V. *et al.* Molecular Mechanism of Switching of TrkA/p75^NTR^ Signaling in Monocrotophos Induced Neurotoxicity. *Sci. Rep.* **5**, 14038; doi: 10.1038/srep14038 (2015).

## Supplementary Material

Supplementary Information
