# Author Correction: Molecular Mechanism of Switching of TrkA/p75^NTR^ Signaling in Monocrotophos Induced Neurotoxicity

**DOI:** 10.1038/s41598-020-63175-5

**Published:** 2020-04-20

**Authors:** Vivek Kumar, Amit Kumar Gupta, Rajendra Kumar Shukla, Vinay Kumar Tripathi, Sadaf Jahan, Ankita Pandey, Akriti Srivastava, Megha Agrawal, Sanjay Yadav, Vinay Kumar Khanna, Aditya Bhushan Pant

**Affiliations:** 1 CSIR-Indian Institute of Toxicology Research, Lucknow, 226001 India; 2 CSIR-Central Drug Research Institute, Lucknow, 226001 India

Correction to: *Scientific Reports* 10.1038/srep14038, published online 15 September 2015

This Article contains an error in Figure 3B, where the flowcytometric image of STS-100nM is a duplication of MCP-100μM. The correct Figure 3B appears below as Fig. 1.

**Figure 1:**
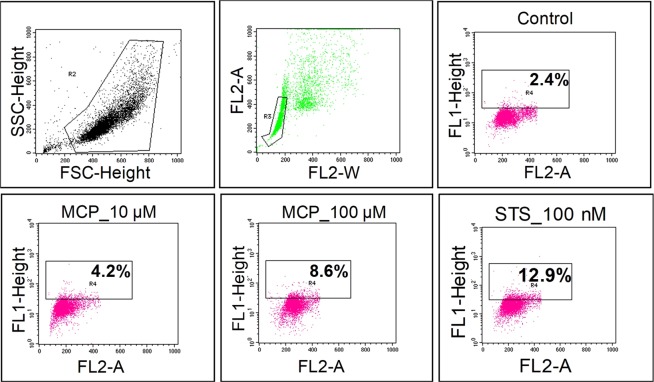
(**B**) DNA damage analysis in stem cell derived neural cells using APO-BrdU™ TUNEL (deoxynucleotide transferase dUTP nick end labeling) Assay Kit with Alexa Fluor® anti-BrdU (Molecular Probes, Invitrogen detection Technologies, USA, Cat No.# A23210) by a flowcytometer (BD-FACS Canto, USA) equipped with BD FACS Diva, version 6.1.2, software. Debris was excluded by forward and side-way light-scattering. (**a**) Control cells, b. MCP-10 μM, c. MCP-100 μM, d. STS-100 nM.

