# Supplementary material for: Molecular Mechanism of Switching of TrkA/p75NTR Signaling in Monocrotophos Induced Neurotoxicity
Source: Sci Rep. 2015 Sep 15;5:14038. doi: 10.1038/srep14038 (PMC4570211; doi:10.1038/srep14038)
Supplement: Supplementary Information [file srep14038-s1.pdf]

Molecular mechanism of switching of TrkA/p75^{NTR} Signaling in Monocrotophos induced neurotoxicity

Vivek Kumar[1], Amit Kumar Gupta[2], Rajendra Kumar Shukla[1], Vinay Kumar Tripathi[1], Sadaf Jahan[1], Ankita Pandey[1], Akriti Srivastava[1], Megha Agrawal[1], Sanjay Yadav[1], Vinay Kumar Khanna[1], Aditya Bhushan Pant[1*]

[1]CSIR-Indian Institute of Toxicology Research, Lucknow-226001, India
[2]CSIR-Central Drug Research Institute, Lucknow-226001, India

Supporting Information

Materials and Methods:

Isolation and characterization of neural stem cells (NSCs)

The tissue was dissected, washed with cold HBSS, minced thoroughly and incubated in 0.1% trypsin for 30 min, then 10 min in DNase (40 µg/ml) at 37 °C. The tissue was then homogenized gently to obtain a single cell suspension. The cells were showing more than 95% viability as determined by trypan blue dye exclusion. The cells were plated at a density of 0.5×10^6 viable cells/ml in 75 cm^2 flasks in serum free neurobasal medium containing N-2 supplement (1%), B-27 supplement (2%), EGF (10 ng/ml), bFGF (10 ng/ml) and 1% antibiotic-antimycotic solution and allowed them to grow as neurospheres at 37°C in 5% CO_2 and 95% air under high humid conditions. Medium was changed twice weekly, while fresh EGF and bFGF were added every day. Small proliferating neurospheres were appeared after 1 week, which came to maturity by day 20. The neurosphere culture was passaged at an interval of 10-12 days by gently triturating the neurospheres and re-plating the single cell suspension of NSCs. The expanding NSCs were labeled with BrdU (1.0 µM) for 24 h. To visualize the proliferating activity of NSCs and to identify the undifferentiated progenitor cells, neurospheres were co-immunostained with anti-nestin monoclonal antibody (mouse, 1:200; a neural stem and progenitor cell specific marker) and anti-BrdU antibody (rabbit, 1:500, proliferating cells marker).

Identification of non-cytotoxic doses of monocrotophos (MCP)

The cells ($0.5X10^4$ cells per well) were seeded in poly-l-lysine coated 96-well culture plates in 5% CO_2 -95% atmospheric air at 37°C. Following 24 h, the medium was replaced with medium containing MCP (1-1000 µM) and plates were incubated for 24-96 h. In the last 4 h of respective

exposure periods, tetrazolium bromide salt (5mg/mL of stock in PBS) was added (10 µl per well in 100 µl). At the end of the incubation period, the reaction mixture was carefully taken out and 200 µl of culture grade DMSO was added to each well. The plates were kept on rocker shaker for 10 min at room temperature and then analyzed at 550 nm using multi well micro plate reader (Synergy HT, Bio-Tek, USA). Unexposed sets were also run under identical conditions and served as basal control, while cells exposed to STS (100 nM) were used as positive control.

Oxidative stress studies

Reactive Oxygen Species (ROS): Intracellular ROS generation was confirmed using fluorescent microscopy by using 2, 7-dichlorodihydrofluorescein diacetate (DCFH-DA; Sigma Aldrich, USA) dye. Briefly, cells (1×10^4/well) were seeded in 4-well chamber slides and allowed to adhere. Following respective exposures, cells were washed twice with PBS, and re-incubated for 30 min in dark in PBS containing DCFH-DA (20 µM). Slides were washed twice again with PBS and mounted for microscopic analysis. Images were taken by using Nikon Eclipse 80i equipped with Nikon DS-Ri1 12.7 megapixel camera. Quantification of fluorescence was done using the image analysis software Leica Qwin 500, and data were expressed as the fold change of unexposed control cells.

Glutathione (GSH): GSH was measured by commercially available kit (Glutathione Assay Kit, Catalog No. 703002; Cayman). After respective exposures, cells were collected through centrifugation at 700 g for 2 min at 4 ^{0}C and lysed in a lysis buffer. The samples were re-centrifuged at 12,000 x g for 10 min at 4 ^{0}C, and supernatants were collected. To estimate the GSH levels, the lysed samples (90 mL/well) were transferred to 96 well black-bottom plates, mixed with a freshly prepared assay cocktail (10 mL), and incubated for 2 h. Thereafter, plates were read at an excitation wavelength of 380 nm and an emission wavelength of 460 nm using a Multiwell Microplate Reader (Synergy HT; Bio-Tek, USA). The standard curve was plotted using the GSH standard supplied in the kit and was used to calculate the experimental values.

Lipid Peroxidation (LPO): LPO was measured by commercially available kit (TBARS Assay Kit, Catalog No.10009055; Cayman). In brief, after respective exposures, cells were collected through centrifugation at 700 g and resuspended in 1ml PBS. The cell suspensions were sonicated three times for five second intervals at 40 V over ice. To estimate the LPO, the homogenate (100 µL) was transferred to 5 ml vial and mixed with a SDS solution (100 µL).

Thereafter, freshly prepared color reagent (4 mL) was added forcefully down side of each vial, and incubated in vigorously boiling water. After one hour, the vials were placed in an ice bath for 10 min to stop the reaction and further centrifuged at 1600xg at 4^0C for 10 min. Finally, sample (150 µL/well) was loaded in 96-well plate from each vial and read at an absorbance wavelength of 530 nm using a Multiwell Microplate Reader (Synergy HT; Bio-Tek). The standard curve was plotted using the MDA standard supplied in the kit and was used to calculate the experimental values.

Mitochondrial membrane potential (MMP): MMP is one of the earliest biomarker that takes place during induction of the oxidative stress and apoptosis. MMP was confirmed using fluorescent microscopy by using JC1 dye. Briefly, cells (1×10^4/well) were seeded in 4-well chamber slides and allowed to adhere. Following respective exposures, cells were re-incubated for 30 min in dark in PBS containing JC1 (1 µM) at 37^0C. Slides were washed twice again with PBS and mounted for microscopic analysis. Images were taken by using Nikon Eclipse 80i equipped with Nikon DS-Ri1 12.7 megapixel camera.

TUNEL assay: TUNEL assay is a method for detecting DNA fragments during the cell cycle by labeling of the deoxynucleotide transferase dUTP nick end. The assay was carried out using APO-BrdU TUNEL Assay Kit with Alexa Fluor 488 anti-BrdU (Molecular Probes, Invitrogen detection Technologies, USA, Catalog No. A23210) by a flowcytometer (BD-FACS Canto, USA) equipped with BD FACS Diva, version 6.1.2, software. Debris was excluded by forward and side-way light-scattering.

Western blot analysis (*In vitro*):

Western blot analysis was conducted following the protocol described earlier [1]. In brief, following respective exposures, cells were scraped, pelleted, and lysed using CelLytic M Cell Lysis Reagent (Sigma) in the presence of protein inhibitor cocktail (Sigma). After protein estimation by Bradford’s Reagent (Fermentas Inc., Glen Burnie, MD), equal amounts (40 µg/ well) of denatured proteins were loaded onto Tricine–SDS/ SDS-PAGE gel according to the protein molecular weight and blotted onto a polyvinylidene fluoride membrane (Millipore) by wet transfer method using transfer buffer (25mM Tris [pH 8.3], 190mM glycine, and 20% methanol) at 250 mA current for 2 h. Nonspecific binding was blocked with 2% BSA and 3% nonfat dry milk powder in TBST (20mM Tris-HCl [pH 7.4], 137mM NaCl, and 0.1% Tween 20)

for 2 h at 37°C. After blocking, the membranes were incubated overnight at 4°C with primary antibodies specific for BCl_2, GAP43 (1:1000, Abcam), p-TrkA, TrkA, $p75^{NTR}$ p-Akt, Akt p-ERK1/2, ERK1/2, p-CREB, NeuN, cytochrome-c, Bax, Caspase 9, caspase 3, ChAT, p-JNK1/2 (1:1000, Chemicon), PARP (1:1000, Cell Signaling Technology), NF-M, (1:1000, Stem Cell Technologies), TUJ-1 and β-actin (1:2000, Sigma) in blocking buffer (pH 7.5). The membrane was then incubated for 2 h at room temperature with secondary anti-primary immunoglobulin G (IgG)–conjugated horseradish peroxidase (Chemicon). The blots were developed using Super Signal West Femto Chemiluminescent Substrate (ThermoFisher Scientific) and Bio-Rad Versa Doc Imaging System 4000 (Bio-Rad, Philadelphia, PA). The densitometry for protein-specific bands was conducted in Gel Documentation System (Alpha Innotech) with the help of Alpha Ease FC Stand Alone V.4.0 software.

Immunocytochemical localization (*In vitro*): Immunocytochemical localization was conducted following the protocol described earlier [2,3]**.** After completion the respective exposures, cells were fixed in 4% paraformaldehyde for 20 min. Cells were then washed with PBS twice and incubated for 15 min in PBS containing 0.02% Triton X 100 and 0.1% BSA to block the nonspecific binding sites. Cells were then washed with PBS and incubated overnight at 4^0C with primary antibodies against specific proteins, viz., NF-M- (1:200), nestin (1:200), ChAT (1:200), TUJ-1 (1:400), BrdU (1:500), $p75^{NTR}$ (1:200),TrkA (1:200); Cytochrome-c (1:50), and Bcl_2 (1:100); all antibodies were from Chemicon International, except Bcl_2 , NF-M and TUJ-1, BrdU, which were procured from Abcam, Stem Cell Technologies and Sigma, respectively). All the antibodies were diluted in PBS containing 0.02% Triton X 100 and 0.1% BSA. Following incubation with primary antibodies, cells were washed three times with PBS for 5 min each to remove the unbound antibodies. Then, secondary antibodies (alexafluor 488 goat anti-mouse IgG (H+L), alexafluor 488 goat anti-rabbit IgG (H+L) and alexafluor® 568 goat anti-rabbit IgG (H+L) (1:400) (Life Technologies)), were added to each well and kept on a rocker shaker in dark for 2 h at room temperature. Cells were then washed with PBS three times for 5 min each and the cell nuclei was counterstained with 4’-6-diamidino-2-phenylindole (DAPI) followed by mounting with anti-fade (Invitrogen) cover slips. Thereafter, the cells were visualized under an upright fluorescence microscope (Nikon Eclipse 80i equipped with Nikon DS-Ri1 12.7-megapixel camera, Japan) using specific filters for FITC and rhodamine. For each marker, 20 randomly selected microscopic fields were captured and analyzed for fluorescence intensity with the help

of Leica Qwin 500 Image Analysis Software (Leica, Germany). The values were expressed in mean ± SE of percent area for fluorescence intensity covered.

I**mmunohistochemistry (*In vivo*):** Immunohistochemical studies were carried out following the method of Ansari et al. [4]. Briefly, rats were anesthetized using ketamine/xylazine (37.5 mg/kg/5 mg/kg body weight, i.p.) and perfused with 150 ml of phosphate-buffered saline (PBS, 0.1 M, pH 7.4) followed by 250 ml of ice-cold 4% paraformaldehyde in PBS for fixation of tissues. Brains were removed and post-fixed in 10% paraformaldehyde in PBS and the samples were kept in 10, 20, and 30% (w/v) sucrose in PBS. Serial coronal sections of 20 µm thickness were cut on a cryomicrotome (Microm HM 520, Labcon, Germany), incubated with primary [anti-TrkA and anti-$p75^{NTR}$, Millipore, USA, 1:200] and secondary antibodies alexafluor 488 goat anti-rabbit IgG (H+L) (1:400) and alexafluor 568 goat anti-rabbit IgG (H+L) (1:400) (Life Technologies,) The intensity of anti-TrkA and anti-$p75^{NTR}$ in the hippocampal region of the brain was determined using a computerized image analysis system (Leica Qwin 500 image analysis software) as described by Shingo et al. [5].

Western blot analysis (*in vivo*): Expressional changes in frontal cortex and hippocampus region of rat brain following the exposure of MCP-were assayed following the method of Yadav et al. [6]. The similar marker proteins were observed for alterations in the expression as seen in the *in vitro* studies. The brain regions were homogenized and proteins were isolated for further processing as per the protocol of Yadav et al. [6]. Rest of the procedure used for western blot analysis was identical as described in the *in vitro* section.

TUNEL assay: DNA fragments were investigated by labeling of the deoxynucleotide transferase dUTP nick end in brain hippocampus section using the APO-BrdU TUNEL Assay Kit (A23210, Molecular Probes, Invitrogen detection Technologies, USA) according to the manufacturer's protocol. Briefly, hippocampus sections were transferred to ice-cold 70 % ethanol overnight, and then rinsed in washing buffer at room temperature and incubated with 50 µl of DNA labelling solution overnight at room temperature. Section were washed twice in rinse buffer and then incubated in 50 µl of freshly prepared antibody labelling solution for 1 h at room temperature. Afterward, the samples were rinsed in PBS and immersed overnight at 4 ^{o} C in a solution containing a monoclonal antibody against Tuj1 (Sigma- Aldrich). The antibody was diluted 1:100 in blocking solution (1 % Triton X-100, 3 % goat serum in PBS). Specimens were rinsed

three times with PBS and incubated for 1 h with a secondary antibody labelled with Alexa Fluor 555 (goat anti-rabbit IgG, Invitrogen) dissolved in PBS (1:400). Slides were analyzed using an inverted fluorescence microscope (Nikon Eclipse 80i equipped with Nikon DS-Ri1 12.7-megapixel camera, Japan) using green fluorescence of Alexa Fluor 488 dye-labeled TUNEL positive cells with DNA fragmentation (excitation 495 nm, emission 519 nm) and red fluorescence of Alexa Fluor 555-labeled neuronal marker TUJ1 (excitation 555 nm, emission 565 nm).

Reference:

1. Kumar, V., *et al.* Lead Intoxication Synergies of the Ethanol-Induced Toxic Responses in Neuronal Cells-PC12. *Mol Neurobiol*, doi:10.1007/s12035-014-8928-x (2014).
2. Tripathi, V.K., *et al.* Monocrotophos induces the expression and activity of xenobiotic metabolizing enzymes in pre-sensitized cultured human brain cells. *PLoS One* **9**, e91946 (2014).
3. Kashyap, M.P., *et al.* Differentiating neurons derived from human umbilical cord blood stem cells work as a test system for developmental neurotoxicity. *Mol Neurobiol* **51**,791–807 (2015).
4. Ansari, R.W., *et al.* Cholinergic dysfunctions and enhanced oxidative stress in the neurobehavioral toxicity of lambda-cyhalothrin in developing rats. *Neurotox Res* **22**, 292-309 (2012).
5. Shingo, T., Date, I., Yoshida, H. & Ohmoto, T. Neuroprotective and restorative effects of intrastriatal grafting of encapsulated GDNF-producing cells in a rat model of Parkinson's disease. *J Neurosci Res* **69**, 946-954 (2002).
6. Yadav, R.S., *et al.* Neuroprotective efficacy of curcumin in arsenic induced cholinergic dysfunctions in rats. *Neurotoxicology* **32**, 760-768 (2011).

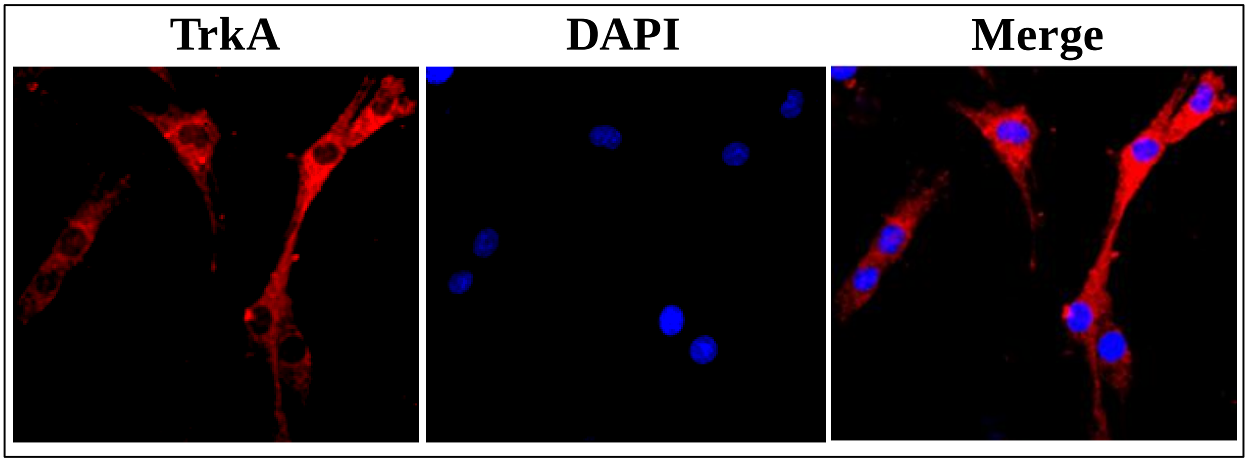

Figure-S1. Photomicrograph showed TrkA positive cells and counter stained with DAPI.

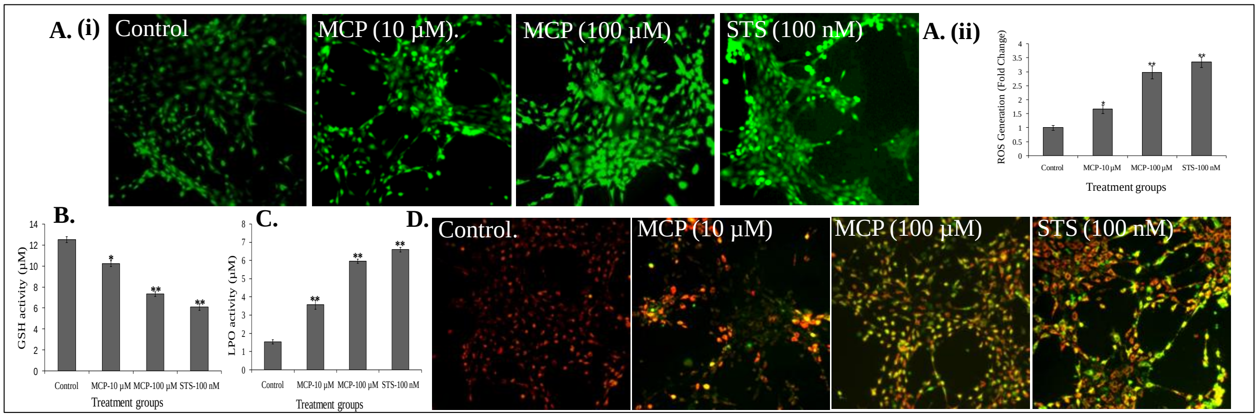

Figure-S2 (A). (i) Increase in the ROS production detected by 2, 7-dichlorodihydrofluorescein diacetate (DCFH-DA) dye under inverted fluorescence microscope. Cells were exposed to MCP (10,100 µM) and STS (100 nM) for 6 h. (ii) Relative quantification expressed in fold induction of ROS generation using Leica Q win500 image analysis Software. **(B).** The altered levels of Glutathione (µM) in cells exposed to MCP (10,100 µM) and STS (100 nM) for 24 h. **(C).** The altered levels of LPO (µM) in cells exposed to MCP (10,100 µM) and STS (100 nM) for 24 h. The values are of derived from three independent experiments an each experiment was having minimum of three replicates. The significance levels are depicted as * =p<0.05; ** p<0.01; *** p<0.001. **(D).** Mitochondrial membrane potential (MMP) was evaluated using JC-1 dye in the cells exposed to MCP (10,100 µM) and STS (100 nM) for 24h. Intact mitochondrial activity is depicted in unexposed control cells (red color), while MCP/ STS exposed cells were having impaired MMP (green color).

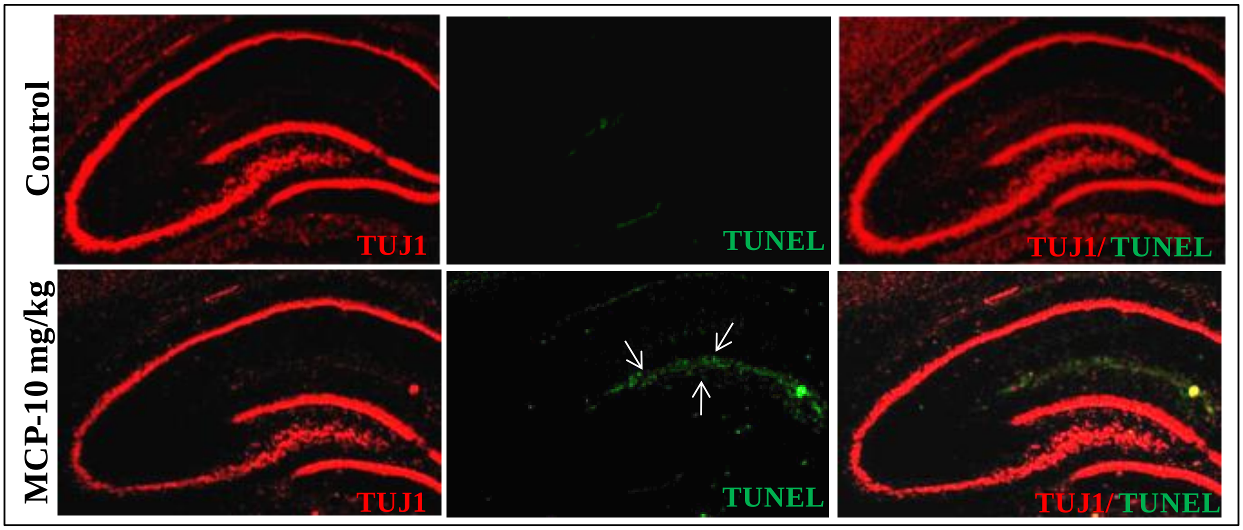

Figure-S3. Photomicrograph of hippocampus after treatment with MCP with an antibody against Tuj1 and secondary antibody labelled with Alexa Fluor 555 (red).Specimens also labelled with TUNEL (green) to identify cells dying by apoptosis. TUNEL positive cells were shown by arrow.

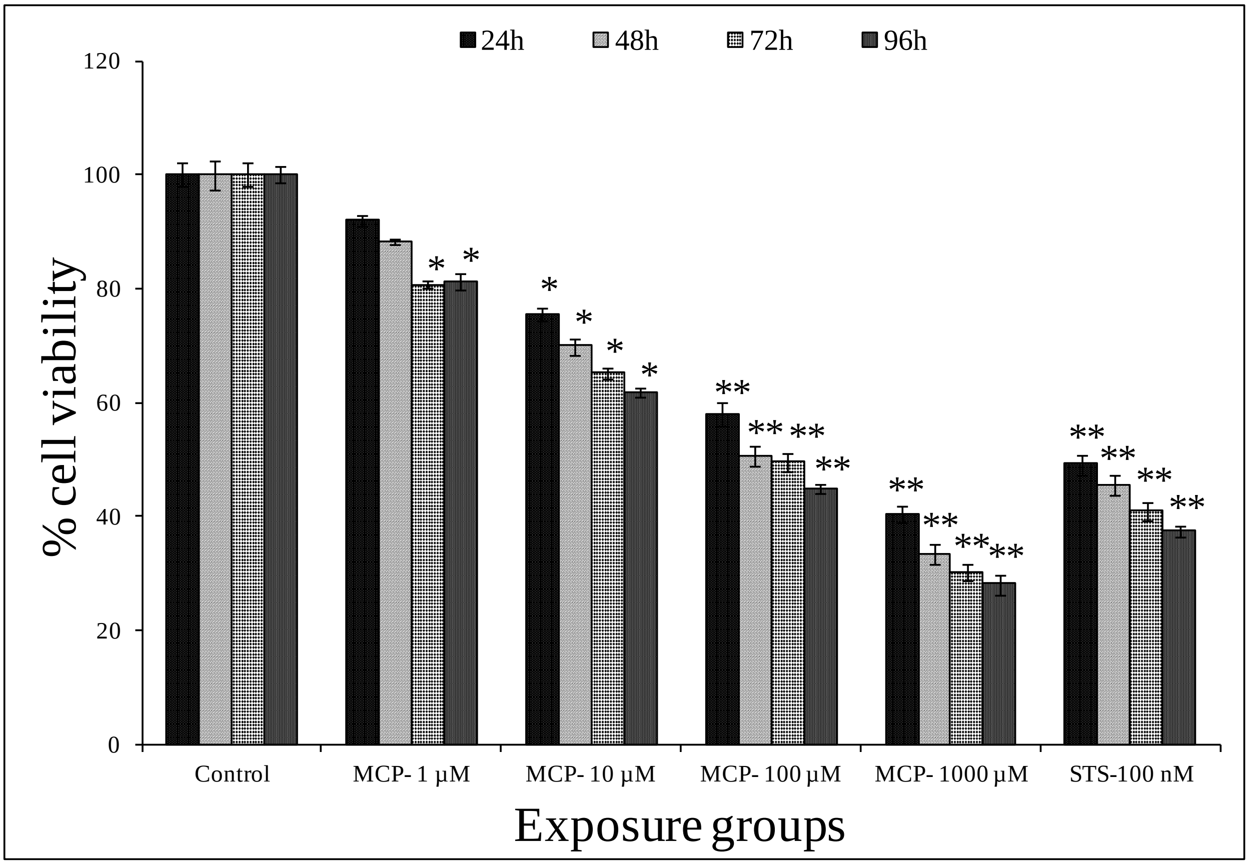

Figure-S4. Identification of biological safe doses of MCP by MTT assay in NSCs cells following the exposure of MCP (0.1 to 1000 µM) or STS nM for 24-96h. * =p<0.05; ** p<0.01 (unexposed control vs experimental group).

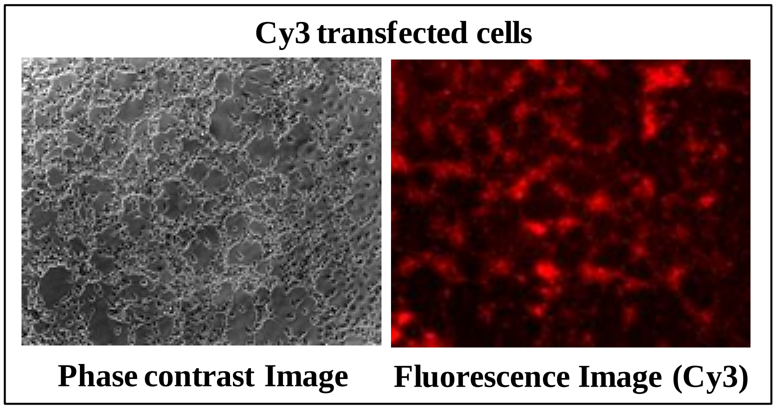

Figure-S5. Transfection efficiency (80 %) was achieved and confirmed by CY3 siRNA (silencer CY3 labeled negative control siRNA, Ambion, AM4621)) using Lipofectamine 2000 (Invitrogen).
